# The comparative burden of brain and central nervous system cancers from 1990 to 2021 between China, the United States, the United Kingdom, and Japan

**DOI:** 10.1186/s12889-025-23982-9

**Published:** 2025-08-06

**Authors:** Zirui Li, Xin Chen, Shuai Han, Anhua Wu

**Affiliations:** 1 Department of Neurosurgery, Shengjing Hospital of China Medical University, No. 36, Sanhao Street, Heping District, Shenyang, 110004 Liaoning China; 2 Institute of Health Sciences, China Medical University, Shenyang, Liaoning China

**Keywords:** Brain and CNS cancers, Epidemiology, Global burden of disease, Disability-adjusted life years, Health inequality, Prediction, China, The united states, The united kingdom, Japan

## Abstract

**Background:**

Brain and central nervous system (CNS) cancers impose a substantial and growing disease burden in China, marked by elevated mortality and disability rates. Understanding disparities in cancer control strategies between China and developed countries (the US, the UK, and Japan) may inform evidence-based policy improvements.

**Methods:**

Using Global Burden of Disease (GBD) 2021 data, we analyzed incidence, prevalence, mortality, and disability-adjusted life years (DALYs) for brain and CNS cancers (1990–2021). Trends were assessed via estimated annual percentage change (EAPC), while frontier analysis evaluated disease burden reduction capacity relative to socioeconomic development. Health inequalities were measured using the Slope Inequality Index (SII) and Concentration Index. Future trends were projected via ARIMA models.

**Results:**

In 2021, China reported 105,541 new cases and 68,911 deaths. While China had lower age-standardized incidence (ASIR) and prevalence (ASPR) rates than comparison nations, its DALYs rate was higher. From 1990 to 2021, ASIR and ASPR increased universally, whereas age-standardized mortality (ASMR) and DALYs (ASDR) declined in China, the US, and the UK but rose in Japan. High-SDI regions exhibited greater burden mitigation capacity, with widening cross-country inequalities. Projections indicate rising ASIR in all nations by 2036.

**Conclusions:**

Despite declining mortality, China’s brain and CNS cancer burden remains disproportionately high. Policymakers should integrate effective strategies from developed nations while tailoring interventions to China’s unique epidemiological and healthcare context.

**Supplementary Information:**

The online version contains supplementary material available at 10.1186/s12889-025-23982-9.

## Introduction

Brain and central nervous system (CNS) cancers represent a heterogeneous group of malignancies with distinct histopathological and molecular features. These neoplasms originate from neuroepithelial tissues (e.g., gliomas), meninges (e.g., meningiomas), and other neural structures, exhibiting a broad spectrum of biological behaviors from indolent (WHO grade I) to highly aggressive (WHO grade IV) phenotypes [1]. Global Cancer Observatory (GLOBOCAN) 2022 data indicate an annual incidence of 321,476 cases (1.6% of total cancers) and 248,305 deaths (2.6% of cancer mortality), with particularly poor 5-year survival rates (33% in high-income countries) [2–4]. The substantial disease burden stems not only from high mortality but also from severe neurological morbidity, including cognitive impairment, motor deficits, and seizures that significantly compromise patients’ quality of life [5, 6].

The pathogenesis of brain and CNS cancers remains poorly understood compared to other malignancies. To date, ionizing radiation exposure represents the only well-established risk factor confirmed through robust epidemiological studies [7, 8]. Other potential associations (e.g., mobile phone use, occupational exposures) remain inconclusive due to inconsistent evidence. This etiological knowledge gap severely constrains primary prevention strategies, necessitating greater emphasis on secondary prevention through early detection and optimized treatment protocols. The development of molecular diagnostics and targeted therapies offers promising avenues for improving outcomes, though significant challenges remain in translating these advances into clinical practice, particularly in resource-limited settings [9, 10].

Significant geographical variations exist in brain and CNS cancer burden, with developing nations demonstrating an emerging epidemiological transition [11]. China presents a unique case study, exhibiting rising ASIR alongside persistently high DALYs. This paradox reflects systemic challenges in early detection, treatment accessibility, and multidisciplinary care coordination [12]. In contrast, developed countries like the US, the UK, and Japan have implemented successful control strategies through NCI-led precision medicine initiatives (the US), NHS-integrated care pathways (the UK), and nationwide cancer registry systems (Japan) [13–19]. These models provide valuable benchmarks for healthcare system improvement in developing nations.

This study employs GBD 2021 data to conduct a comprehensive comparative analysis of brain and CNS cancer epidemiology across China and three benchmark countries (the US, the UK, Japan). Using advanced analytical methods including autoregressive integrated moving average (ARIMA) modeling for projections (2022–2036), we quantify temporal trends in incidence, prevalence, mortality, and DALYs. The study incorporates frontier analysis to assess health system efficiency and inequality indices (SII, Concentration Index) to evaluate disparities. Findings will provide evidence-based recommendations for optimizing China’s cancer control policies through strategic adoption of international best practices, with particular focus on strengthening early detection systems, specialized care networks, and palliative care integration.

## Methods

### Data sources

The Global Burden of Disease (GBD) Study 2021 comprehensively assessed health loss caused by 371 diseases and 88 risk factors in 204 countries and regions using the latest epidemiological data sources and improved standardized methods [20]. It provides a comprehensive framework for assessing global disease burden. The data were systematically sourced from established public databases following rigorous screening to ensure data quality. The GBD authors commit to annual updates for accuracy [3]. The data collected by the GBD collaborative network underwent standardized cleaning, transformation, and modeling processes implemented by research organizations worldwide to produce robust estimates. In the present study, we collected GBD Study data on brain and CNS cancers incidence, prevalence, mortality, and DALYs from 204 countries and regions. Patients age was divided into 20 subgroups at every 5-year interval. The data can be found on the website of the Institute of Health Measurement and Evaluation (https://ghdx.healthdata.org/gbd-2021/sources).

Socio-demographic index (SDI) is a composite index to assess the development status of a country or region. It integrates three key elements: national-level income per capita, average years of schooling, and the total fertility rate (TFR) in females under the age of 25 years [21]. SDI values range from 0 to 1, with values closer to 0 indicating lower socioeconomic progress and values approaching 1 reflecting advanced development across all SDI components.

### Statistical analysis

In this study, we use age-standardized rates (ASRs) for incidence, prevalence, mortality, and DALYs, as well as estimated annual percentage change (EAPC) to assess the disease burden of brain and central nervous system cancers. The Estimated annual percentage change (EAPC) is commonly used to measure trends of age-standardized rates over a period of time. A linear fit to the log-transformed ASR is performed with year as the independent variable, y = α + βx + ε, y = ln (ASR), x = year. The EAPC and its 95% CI were calculated as EAPC = 100 ×[exp(β) − 1] using a generalized linear regression model, which represents the annual percentage change [22]. If both EAPC and the 95% CI are greater than 0, it means that the ASR is increasing from year to year, and, conversely, it is decreasing from year to year.

Frontier analysis was used to further evaluate the relationship between brain and CNS cancers burden and sociodemographic development. In order to generate a nonlinear frontier, this frontier implies the minimum achievable burden corresponding to development status. We employed non-parametric data envelope analysis with reference to previous studies [23, 24]. The effective difference is defined as the distance between the ASDR in a country and its frontier, which reflects the unfulfilled health gains that exist based on the current level of development in the country or region.

We extracted total DALYs and age standardized DALY rates (ASDR) for inequality analysis. There are two standard measures, which are Slope Index of Inequality (SII) and Concentration Index, used to assess the inequalities between countries. We used robust linear regression models to calculate SII and its 95% UI. SII represents the slope of the regression line between the ASDR and the weighted ranking of each country. The Relative Index of Inequality (RII) is obtained by dividing SII by ASDR. Lorenz concentration curves were fitted according to cumulative DALYs and cumulative population to assess the relative differences in the burden of brain and CNS cancers among countries. The Concentration Index is a numerical integration of the area under the curve, ranging from − 1 to 1. A positive Concentration Index value indicates a higher concentration of brain and CNS cancers burden among populations living in countries with higher SDI [25].

The ARIMA is a common forecasting method in epidemiology to predict trends by analyzing time series of historical data which consists of the autoregressive (AR) model and moving average (MA) model [26]. The underlying assumption is that data series are time-dependent random variables, whose autocorrelation can be characterized by the ARIMA model, and future values can be predicted based on past values. The equation is expressed as Yt = φ1Yt − 1 + φ2Yt − 2 +. + φpYt − p + et − θ1et − 1 −. − θqet − q, where (φ1Yt − 1 + φ2Yt − 2 +. + φpYt − p + et) is the AR model part, et − θ1et − 1 −. − θqet − q is the MA model part, Yt − p is the observed value at the period of (t − p), p and q represent the model order of AR and MA, and et is the random error at the period of t [27]. The time series in the ARIMA model should be a stationary and stochastic sequence with zero mean. Based on the GBD database of 2021, we applied R language to construct an ARIMA model to predict the global cancer burden of the brain and central nervous system of four countries from 2021 to 2036.

All data were collated, analyzed and plotted using R software (version 4.3.1), and *p* < 0.05 was considered statistically significant.

## Results

### Global burden of brain and CNS cancers: geographical disparities in 2021

**Fig. 1:**
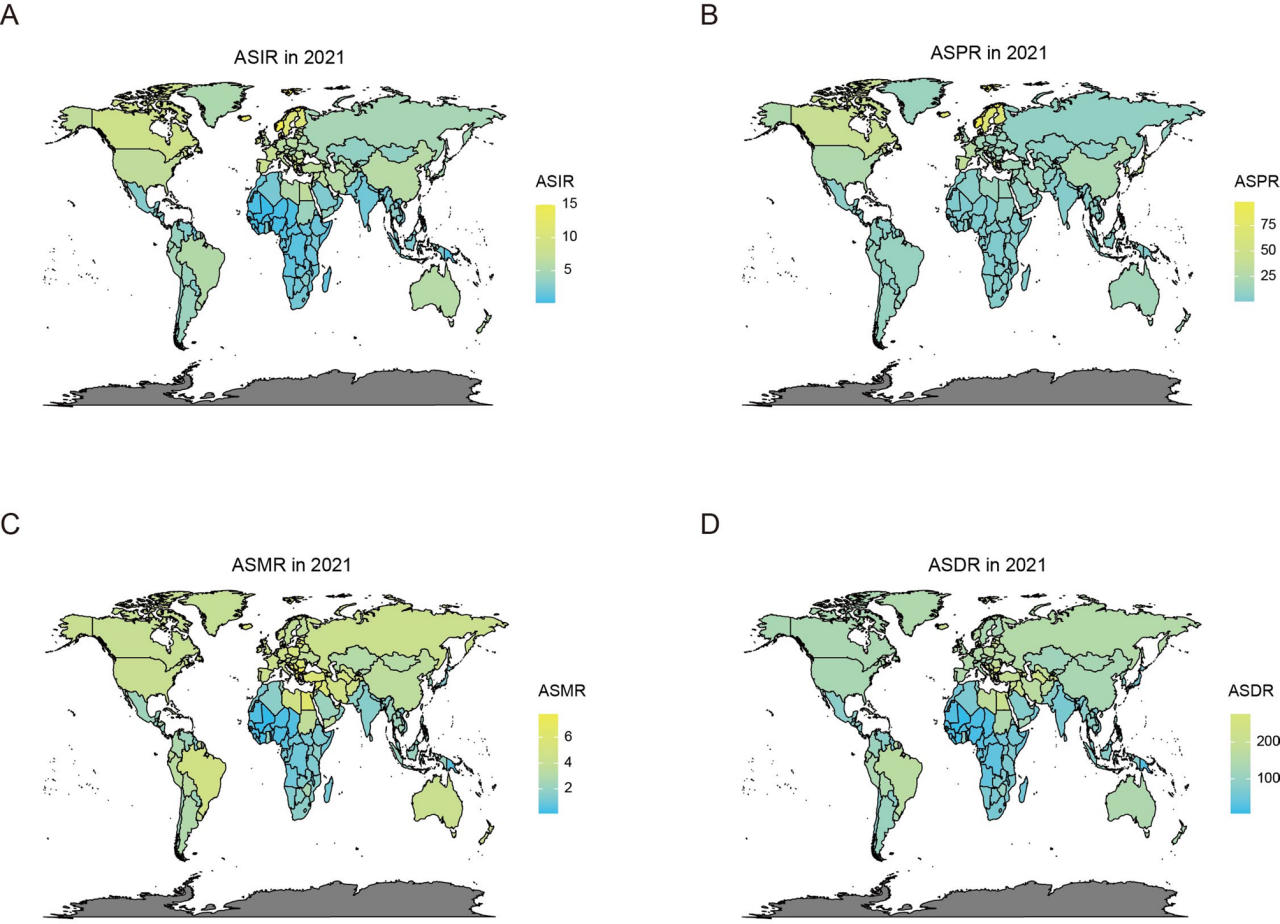
Map presentation of global ASIR, ASPR, ASMR, and ASDR for brain and CNS cancers, 2021. **A** ASIR for brain and CNS cancers in 204 countries and territories in 2021; **B** Distribution of ASPR of brain and CNS cancers globally, 2021; **C** Map showing ASMR for brain and CNS cancers in 204 countries and territories in 2021; **D** ASDR of brain and CNS cancers in 2021 in 204 countries and territories

Our analysis of 204 countries revealed significant geographical variations in brain and CNS cancer burden (Fig. 1). Norway exhibited the highest age-standardized prevalence (ASPR: 98.3 per 100,000) and incidence rates (ASIR: 15.0 per 100,000), while Gambia showed the lowest values (ASPR: 0.3 per 100,000; ASIR: 0.1 per 100,000). Mortality patterns followed similar trends, with Montenegro demonstrating the highest age-standardized mortality rate (ASMR: 7.8 per 100,000) and Gambia the lowest (ASMR: 0.1 per 100,000). China bore the greatest burden, accounting for 29.5% of global cases (105,541/357,482) and 26.6% of deaths (68,911/258,627). Comparatively, the US, the UK and Japan contributed substantially lower proportions (incidence: 8.9%, 1.7%, 3.2%; mortality: 8.3%, 1.9%, 1.2% respectively). These findings highlight significant international disparities in brain and CNS cancer epidemiology.

### Trends in brain and CNS cancers burden in China, the US, the UK, and Japan

Analysis of GBD 2021 data revealed substantial increases in brain and CNS cancer burden across all four studied nations between 1990 and 2021 (Table 1). China experienced the most dramatic growth, with prevalent cases increasing 2.8-fold from 108,115 (95% UI: 77,294 − 130,965) to 305,063 (244,871 − 387,362), while incident cases more than doubled from 47,364 to 105,541. The US showed more moderate growth, with prevalent cases rising from 55,754 to 84,402. Mortality trends followed similar patterns, with China’s deaths increasing from 39,160 to 68,911, compared to the US’s rise from 13,247 to 21,444. Disability burden, measured in DALYs, grew substantially in all countries, with China’s DALYs increasing by 19.3% to reach 2,247,659 (1,715,820-2,880,771) by 2021.

**Table 1:** The disease burden of brain and CNS cancers across four countries in 1990 and 2021

	Case Number (95%UI)			Age-standardized Rates per 100,000 people (95%UI)		
Global burden of brain and central nervous system cancers in 1990						
China						
	Total	Male	Female	Total	Male	Female
Prevalence	108,115 (77294,130965)	54,037 (31850,76791)	54,078 (36008,66611)	9.68 (6.94,11.76))	9.40 (5.52,13.36)	10.02 (6.69,12.32)
Incidence	47,364 (34333,59067)	24,958 (14599,36143)	22,406 (15753,27470)	4.69 (3.42,5.85)	4.90 (2.87,7.06)	4.51 (3.21,5.50)
Deaths	39,160 (28521,49195)	21,441 (12671,30841)	17,719 (12648,21737)	4.05 (2.97,5.04)	4.42 (2.63,6.28)	3.71 (2.68,4.55)
DALYs	1,884,413 (1333559,2298059)	1,053,754 (617586,1496183)	830,659 (547270,1016867)	174.36 (123.93,213.51)	188.87 (112.12,268.70)	159.52 (105.82,194.61)
United states of America						
	Total	Male	Female	Total	Male	Female
Prevalence	55,754 (54576,56911)	29,455 (28712,30110)	26,299 (25441,27119)	21.59 (21.12,22.04)	23.4 (22.83,23.96)	19.98 (19.37,20.65)
Incidence	19,756 (19189,20173)	10,604 (10347,10797)	9152 (8774,9420)	7.01 (6.83,7.14)	8.13 (7.93,8.28)	6.07 (5.89,6.23)
Deaths	13,247 (12788,13529)	7264 (7090,7392)	5983 (5664,6160)	4.48 (4.34,4.56)	5.47 (5.34,5.57)	3.65 (3.5,3.74)
DALYs	435,839 (427480,442709)	247,563 (242742,251793)	188,275 (182458,191919)	159.54 (156.74,161.86)	191.11 (187.38,194.32)	131.30 (128.18,133.45)
United Kingdom						
	Total	Male	Female	Total	Male	Female
Prevalence	9409 (9200,9592)	5190 (5073,5310)	4219 (4073,4366)	15.33 (14.99,15.66)	17.24 (16.81,17.66)	13.53 (13.07,14.04)
Incidence	4187 (4106,4253)	2353 (2311,2395)	1834 (1778,1873)	5.91 (5.81,6.00)	7.05 (6.92,7.18)	4.9 (4.78,5.00)
Deaths	3354 (3285,3398)	1907 (1876,1935)	1447 (1399,1473)	4.46 (4.39,4.51)	5.51 (5.42,5.59)	3.55 (3.47,3.60)
DALYs	110,976 (109466,112258)	64,977 (64098,65879)	45,999 (45047,46613)	168.42 (166.48,170.29)	204.15 (201.42,207.04)	134.96 (133.03,136.6)
Japan						
	Total	Male	Female	Total	Male	Female
Prevalence	24,504 (23319, 25662)	13,111 (12397, 13836)	11,393 (10609, 12205)	17.93 (17.02, 18.89)	20.14 (18.95, 21.3)	15.94 (14.78, 17.19)
Incidence	3944 (3765, 4115)	2153 (2053, 2269)	1790 (1674, 1899)	2.82 (2.69, 2.95)	3.27 (3.09, 3.44)	2.43 (2.28, 2.61)
Deaths	1313 (1271, 1339)	733 (716, 748)	580 (553, 597)	0.89 (0.86, 0.91)	1.06 (1.04, 1.08)	0.74 (0.71, 0.76)
DALYs	52,452 (51297, 53445)	30,078 (29410, 30769)	22,373 (21717, 22935)	40.55 (39.75, 41.28)	47.21 (46.17, 48.3)	34.24 (33.45, 35.01)
Global burden of brain and central nervous system cancers in 2021						
China						
	Total	Male	Female	Total	Male	Female
Prevalence	305,063 (244871, 387362)	135,721 (83916, 192626)	169,342 (129037, 223880)	21.23 (16.98, 26.56)	18.83 (11.73, 27.24)	23.77 (18.58, 30.84)
Incidence	105,541 (81401, 133527)	54,097 (31975, 76036)	51,444 (38738, 67694)	6.12 (4.76, 7.67)	6.33 (3.77, 8.87)	5.96 (4.54, 7.67)
Deaths	68,911 (52055, 88280)	38,403 (22701, 53965)	30,508 (22401, 39569)	3.63 (2.74, 4.60)	4.18 (2.45, 5.77)	3.13 (2.36, 4.07)
DALYs	2,247,659 (1715820, 2880771)	1,289,490 (786067, 1824421)	958,169 (735987, 1275246)	134.15 (102.90, 171.51)	152.81 (94.68, 214.83)	115.15 (89.58, 148.89)
United states of America						
	Total	Male	Female	Total	Male	Female
Prevalence	84,402 (80959, 87131)	45,640 (43802, 47232)	38,762 (36889, 40441)	23.38 (22.43, 24.28)	25.51 (24.32, 26.53)	21.39 (20.33, 22.52)
Incidence	31,780 (29971, 32844)	17,460 (16654, 18045)	14,320 (13269, 14930)	6.91 (6.58, 7.12)	7.96 (7.64, 8.22)	5.97 (5.66, 6.21)
Deaths	21,444 (20046, 22167)	12,132 (11497, 12525)	9312 (8488, 9745)	4.10 (3.87, 4.22)	4.99 (4.76, 5.14)	3.32 (3.09, 3.45)
DALYs	594,996 (571295, 610278)	344,129 (330882, 353859)	250,867 (237588, 259575)	134.38 (129.83, 137.95)	160.70 (155.05, 165.07)	109.90 (105.59, 113.56)
United Kingdom						
	Total	Male	Female	Total	Male	Female
Prevalence	14,468 (13854, 14996)	7923 (7590, 8234)	6545 (6139, 6913)	18.08 (17.43, 18.74)	20.23 (19.47, 21.06)	16.02 (15.21, 16.84)
Incidence	6230 (5880, 6440)	3531 (3387, 3651)	2700 (2492, 2821)	6.20 (5.94, 6.39)	7.31 (7.04, 7.55)	5.16 (4.89, 5.35)
Deaths	4923 (4632, 5102)	2857 (2741, 2953)	2065 (1892, 2153)	4.44 (4.25, 4.59)	5.44 (5.25, 5.61)	3.53 (3.32, 3.65)
DALYs	135,672 (130459, 139584)	80,245 (77668, 82765)	55,427 (52551, 57189)	147.27 (142.67, 151.29)	178.80 (173.45, 184.46)	117.25 (112.81, 120.63)
Japan						
	Total	Male	Female	Total	Male	Female
Prevalence	70,806 (62041, 77106)	38,389 (34445, 41663)	32,417 (26248, 36898)	34.72 (32.05, 37.34)	40.29 (36.70, 43.86)	29.46 (25.89, 32.52)
Incidence	11,323 (9841, 12337)	6229 (5633, 6787)	5093 (4099, 5852)	5.11 (4.74, 5.46)	6.06 (5.56, 6.58)	4.24 (3.78, 4.66)
Deaths	3299 (2967, 3483)	1912 (1798, 1983)	1387 (1164, 1514)	1.38 (1.31, 1.43)	1.70 (1.64, 1.76)	1.09 (1.00, 1.14)
DALYs	92,386 (86533, 96449)	54,955 (52861, 56890)	37,431 (33670, 39695)	58.47 (56.40, 60.27)	69.88 (67.65, 72.26)	47.24 (45.02, 49.02)

**Fig. 2:**
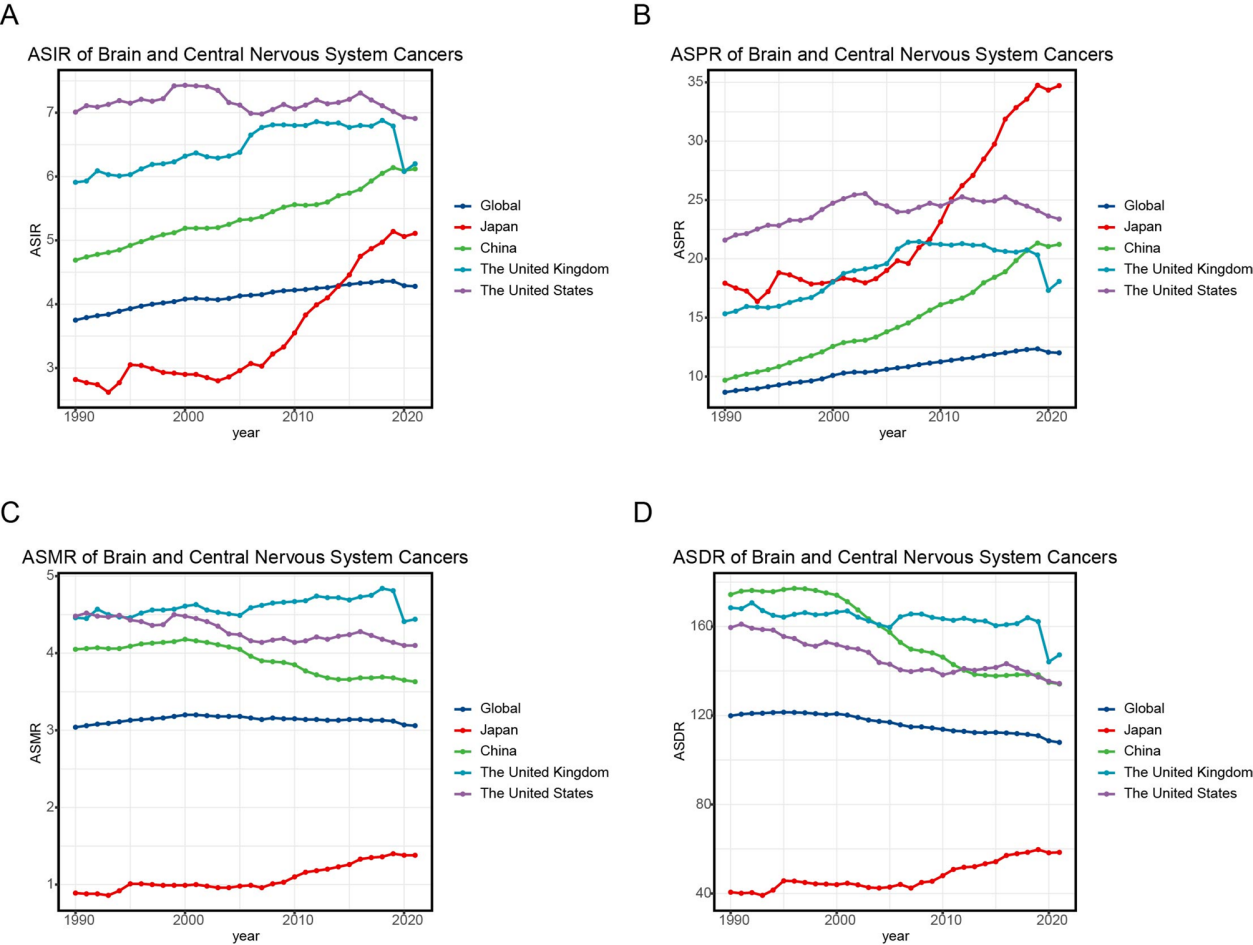
Comparison of temporal changes in brain and CNS cancers burden in four countries. **A** Trends in ASIR for brain and CNS cancers in China, the US, the UK, and Japan from 1990 to 2021; **B** ASPR of brain and CNS cancers by country from 1990 to 2021; **C** Temporal trends for ASMR of brain and CNS cancers in four countries from 1990 to 2019; **D** ASDR for brain and CNS cancers in four countries, 1990 to 2021

Incidence patterns varied notably (Fig. 2A), with China (EAPC = 0.8), the UK (EAPC = 0.4), and Japan (EAPC = 2.2) showing increases, while the US demonstrated a slight decline (EAPC=−0.1). ASPR showed universal increases (Fig. 2B), with China exhibiting the steepest growth (EAPC = 2.7), followed closely by Japan (EAPC = 2.6). Mortality trends revealed divergent pathways (Fig. 2C): China (EAPC=−0.5) and the US (EAPC=−0.3) achieved reductions, contrasting with increases in the UK (EAPC = 0.2) and Japan (EAPC = 1.5). Disability rates showed similar patterns (Fig. 2D), with China demonstrating the most significant improvement (EAPC=−1.1), while Japan’s ASDR increased (EAPC = 1.3). These differential trends highlight varying trajectories in cancer control effectiveness across development contexts.

### Sex and age-specific patterns in brain and CNS cancer burden

**Fig. 3:**
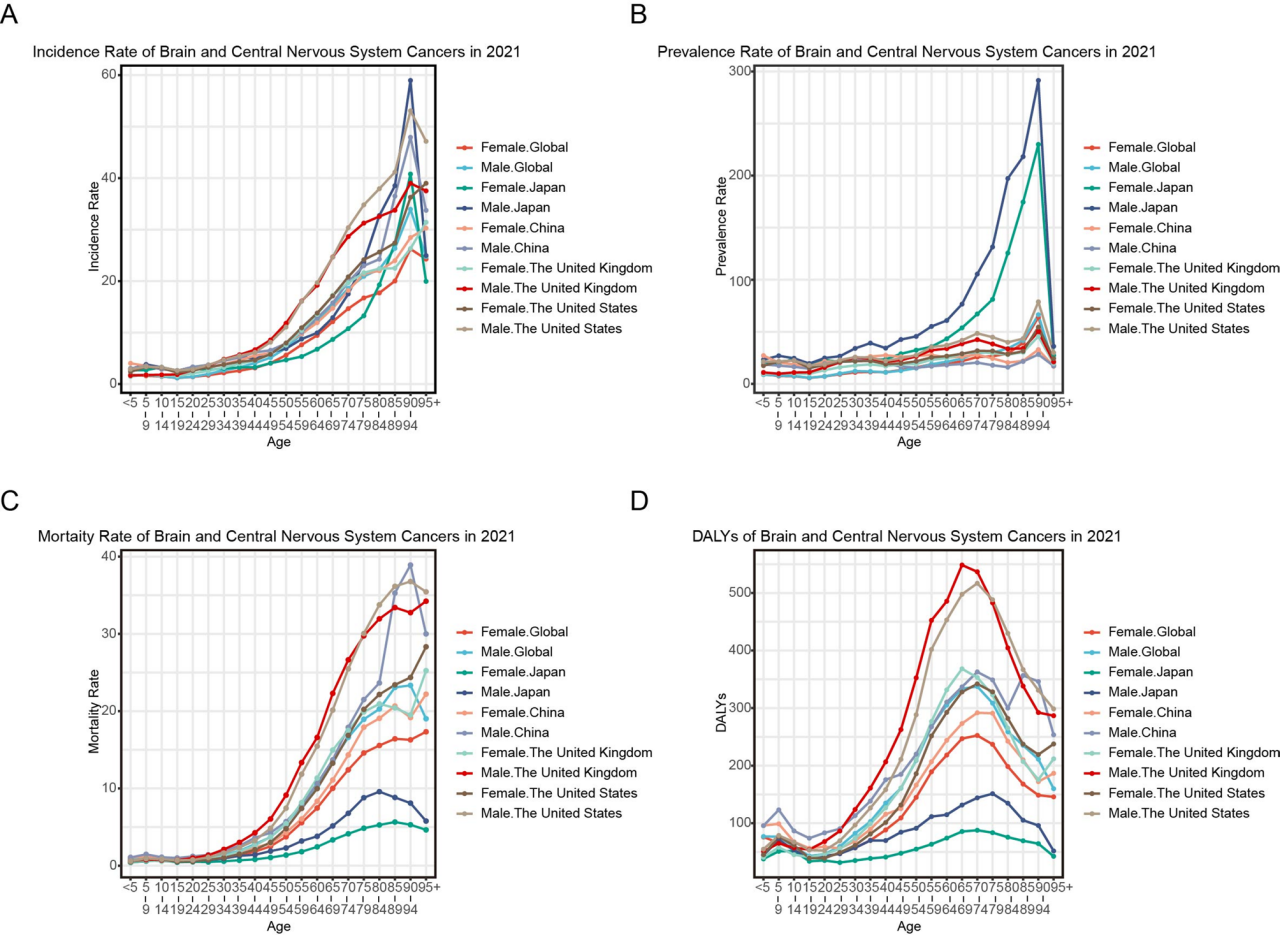
Comparison of brain and CNS cancers burden in different age groups in four countries, 2021. **A** Incidence rate attributable to brain and CNS cancers by age and gender in China, the United States, the United Kingdom, and Japan in 2021; **B** Prevalence rate by age group including both sexes in four countries, 2021; **C** Mortality rate of brain and CNS cancers for each age group by sex in four countries in 2021; **D** Rate of DALYs for brain and CNS cancers by age and sex in four countries, 2021

Our analysis revealed significant sex disparities in brain and CNS cancer burden across all four nations in 2021. Males consistently demonstrated higher incidence, mortality, and DALY rates compared to females (Fig. 3). Notably, this male predominance extended to prevalence counts and age-standardized rates in developed nations (the US, the UK, Japan), while China showed an exception to this pattern. These findings suggest potential biological or environmental factors contributing to sex-specific susceptibility, particularly in developed healthcare systems.

Age-specific analysis demonstrated distinct epidemiological patterns. Prevalence rates followed a monotonic increase with age, peaking at 90–94 years across all populations (Fig. 3B). Incidence and mortality rates showed similar age-related trends, though with some variation in peak age groups by sex and country. Most remarkably, DALY distributions exhibited consistent bimodality across all nations, with primary peaks occurring between 65 and 79 years (varying by country and sex) and secondary peaks at 5–9 years (Fig. 3D). This pattern suggests distinct etiological mechanisms operating at different life stages, possibly reflecting differences in tumor biology between pediatric and adult cases.

### Association between SDI and global burden of brain and CNS cancers

**Fig. 4:**
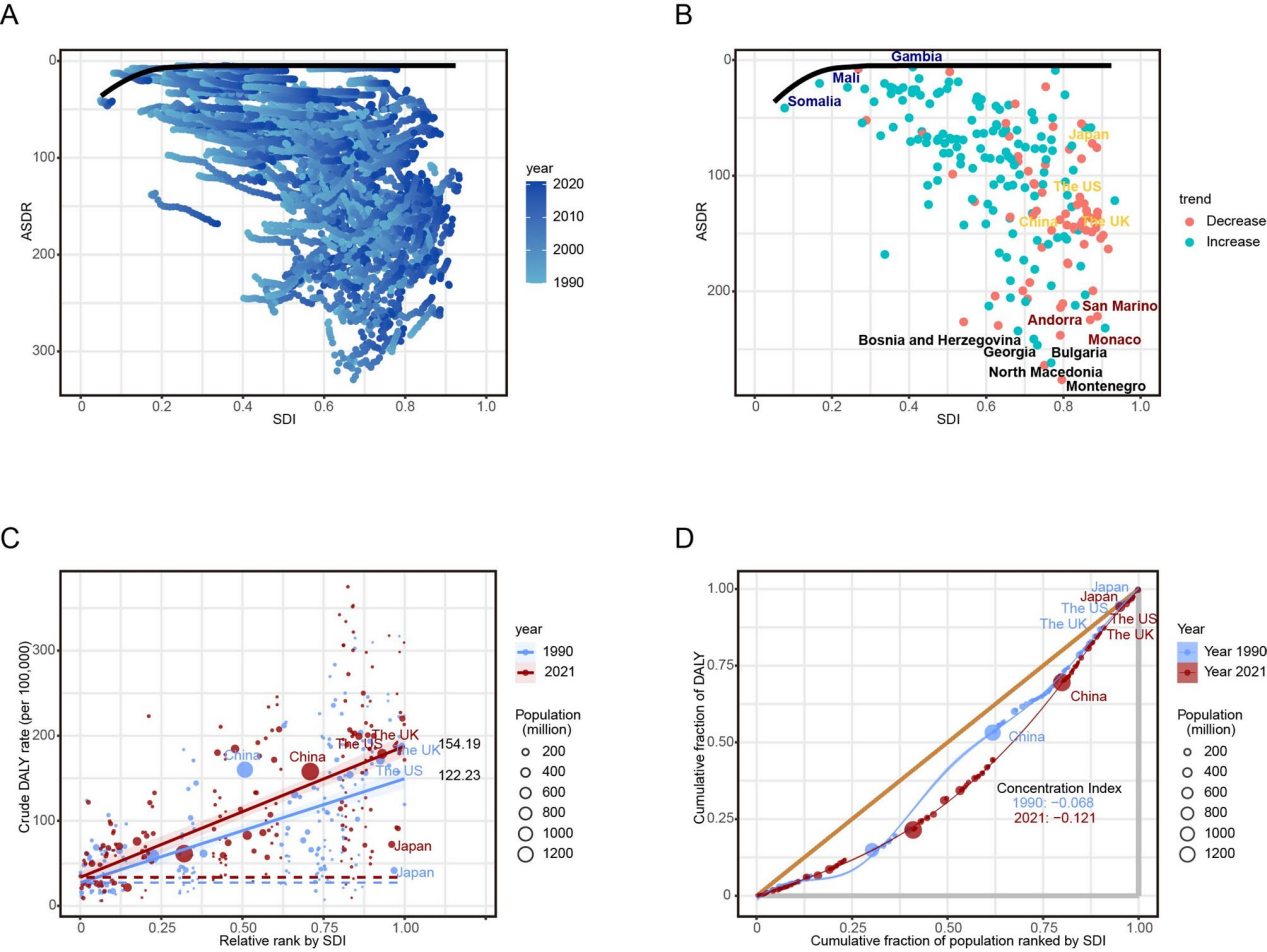
Frontier analysis and health inequality analysis. **A** Frontier analysis based on SDI and ASDR for brain and CNS cancers in 204 countries from 1990 to 2021; **B** The trend of ASDR and frontier analysis of SDI with ASDR for brain and CNS cancers in 204 countries from 1990 to 2021; **C** SII analysis. Absolute income-related healthy inequality in brain and CNS cancers burden in 1990 and 2021; **D** Concentration index analysis. Relative income-related healthy inequality in brain and CNS cancers burden in 1990 and 2021

Frontier analysis was conducted to evaluate the relationship between the SDI and ASDR for brain and CNS cancers across 204 countries and regions from 1990 to 2021. The frontier, represented by a solid black line, demarcates the optimal performance boundary, with individual countries plotted as data points. Red and blue points signify nations with increasing and decreasing ASDR trends, respectively. Notably, the five countries exhibiting the largest effective differences—Monaco, San Marino, and Andorra (high-SDI, marked in red) versus Somalia, Mali, and Gambia (low-SDI, highlighted in blue)—demonstrated significant disparities in ASDR relative to the frontier. China, the US, the UK, and Japan were annotated for comparative analysis. The findings reveal a positive association between SDI and brain and CNS cancer burden, with higher-SDI countries experiencing elevated ASDR (Fig. 4A-B).

Health inequality analysis further corroborated these trends, as measured by the SII for DALYs per 100,000 population. The SII increased from 122.2 (95% UI: 96.1–148.3) in 1990 to 154.2 (95% UI: 128.0–180.3) in 2021, indicating a widening disparity in disease burden between high- and low-SDI nations. This suggests that socioeconomic development is associated with an escalating and unequal distribution of brain and CNS cancer morbidity and mortality (Fig. 4C-D).

### Prediction of brain and CNS cancers burden in China, the US, the UK, and Japan for the next 15 years

**Fig. 5:**
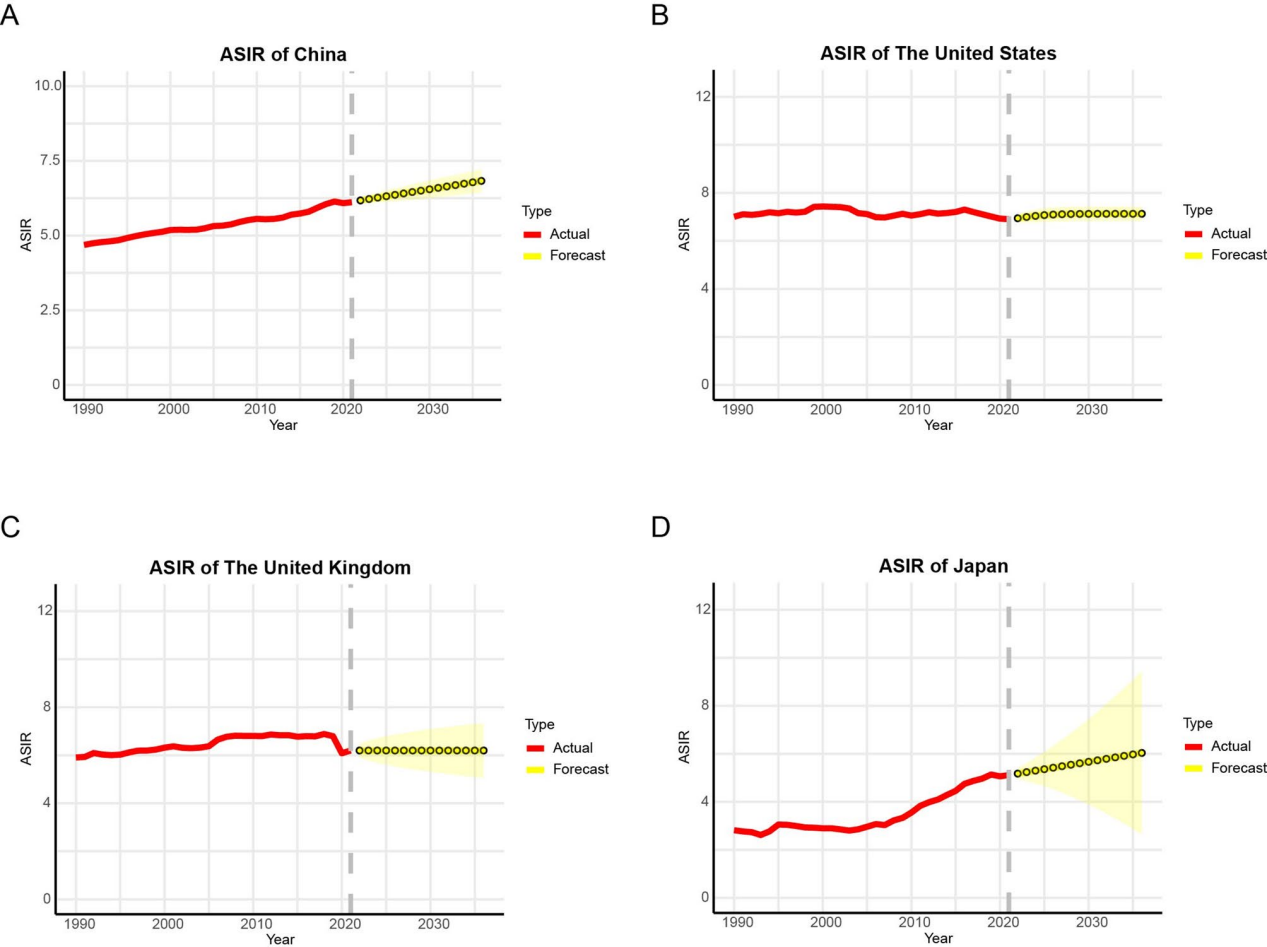
Predictive analysis of ASIR in four countries over the next 15 years. **A** Temporal trend of ASIR for brain and CNS cancers in China from 2022 to 2036; **B** ASIR forecast for brain and CNS cancers in the United States over the next 15 years; **C** Projected temporal trends in ASIR of brain and CNS cancers in the United Kingdom from 2022 to 2036; **D** Temporal trend in ASIR for brain and CNS cancers in Japan over the next 15 years

Using ARIMA modeling, we projected ASIR, ASPR, ASMR, and ASDR rates for brain and CNS cancers in China, the US, the UK, and Japan from 2022 to 2036. Results indicate divergent epidemiological trajectories across these nations. While all four countries are expected to experience rising ASIR (China: 6.8; the US: 7.1; the UK: 6.2; Japan: 6.0 by 2036) (Fig. 5), ASPR patterns show geographical variation, with increases projected in China (27.0) and Japan (42.0) but decreases anticipated in the US (18.7) and the UK (12.9) (Fig. S1). Mortality trends reveal further complexity, with China and the US demonstrating declining ASMR (3.6 and 4.1 respectively), contrasting with increases in the UK (4.4) and Japan (1.6) (Fig. S2). Similarly, ASDR is projected to decrease in China (131.1), the US (122.2), and the UK (147.2), while Japan shows an opposing trend (67.1) (Fig. S3). These forecasts highlight significant international disparities in the future burden of brain and CNS malignancies, suggesting differential impacts of demographic transitions, diagnostic practices, and healthcare systems across these high-income nations.

## Discussion

This study presents the first comprehensive analysis of the burden of brain and CNS cancers in China, the US, the UK, and Japan, leveraging data from the 2021 GBD study. It provides detailed estimates of incidence, prevalence, mortality, and DALYs, alongside an examination of global distribution patterns. The findings reveal that China has a lower ASIR compared with the US and the UK, as well as a lower ASPR relative to all three developed countries. However, China exhibits a higher ASMR than Japan and a higher ASDR compared with both the US and Japan. Significant regional disparities in disease burden were observed, with increasing inequities in distribution. Projections from 2022 to 2036 indicate a rising burden of brain and CNS cancers in all four countries.

The 2021 GBD study’s comprehensive analysis highlighted complex disparities in disease burden and SDI across regions. Norway exhibited the highest ASPR and ASIR, whereas Montenegro had the highest ASMR and ASDR. Gambia displayed the lowest values for all metrics. Countries with higher socioeconomic development demonstrated elevated ASIR but reduced ASMR and ASDR, potentially attributable to superior healthcare quality and resource allocation [28]. China accounted for nearly one-third of global new cases and over one-quarter of global deaths from brain and CNS cancers, underscoring the significant burden on its healthcare system. Targeted public health interventions are crucial to alleviate this growing burden.

From 1990 to 2021, ASPR in China, the US, and the UK increased, while overall ASMR and ASDR declined, which may be explained by advancements in early diagnostics and therapeutic innovations. Japan, however, demonstrated upward trends in all four metrics, a pattern potentially associated with the long-term effects of the 1945 atomic bombings [29]. The UK exhibited a sharp decline in disease burden metrics in 2019, a phenomenon likely attributable to COVID-19 pandemic [4, 30].

China’s lower ASIR and ASPR compared with those of developed countries may be attributed to insufficient early screening awareness and delayed adoption of novel treatments [31, 32]. Despite these challenges, China has made significant strides in reducing ASMR and ASDR, reflecting improvements in surgical techniques and postoperative monitoring [33–35]. The US, the UK, and Japan maintain lower ASDR than China, benefiting from superior medical resources and healthcare accessibility [36].

In 2021, males in all four countries exhibited higher incidence, mortality, and DALYs rates for brain and CNS cancers than females, indicating a greater disease burden among males. However, in China, females exhibited a higher prevalence rate but lower incidence, mortality, and DALYs rates, suggesting improved access to medical resources and heightened awareness of gender equality [37].

DALYs for brain and CNS cancers in 2021 showed a bimodal distribution, with peaks observed in pediatric and geriatric populations, emphasizing the necessity of age-specific preventive and therapeutic strategies. China’s higher pediatric burden compared with developed countries underscores the urgency for enhanced early screening and treatment. Environmental and parental factors during the reproductive period are significant risk factors for pediatric cancers, necessitating minimized exposure to carcinogens and improved neonatal environments [38–40].

In China, the age group with the highest DALYs rate for brain and CNS cancers is younger than that in developed countries, indicating the need for tailored prevention strategies focusing on younger populations. Furthermore, China should prioritize medical resource allocation to middle-aged and elderly patients, who account for a larger proportion of cases compared with developed countries.

Frontier analysis revealed that effective differences in brain and CNS cancers burden generally increase with national development levels, indicating that countries with higher SDI possess greater potential for burden reduction. The inequality in disease burden distribution has intensified since 1990, reflecting a persistent global health challenge. The markedly uneven global distribution of neurological professionals, with high-income countries having significantly more resources, underscores the urgency of cultivating a specialized neurological workforce [41].

Projections for the next 15 years indicate rising ASIR across all four countries, with ASPR increasing in China and Japan but declining in the US and the UK. ASMR are expected to decrease in China and the US but increase in the UK and Japan. ASDR will trend downward in all countries except Japan. China’s relatively rapid ASIR growth and slower reductions in ASMR and ASDR relative to the US and the UK highlight significant disparities in prevention and management capabilities. Adopting successful practices from developed countries, such as cutting-edge diagnostic and treatment technologies, accelerating research on novel anti-cancer drugs, and strengthening multidisciplinary collaboration, is critical for China [42].

Study limitations include reliance on modeled data from the 2021 GBD study, potential misclassification and miscoding, and the aggregation of all brain and CNS cancers into a single group in the GBD database. The absence of detailed epidemiological data and risk factor analysis limits the precision of the study. Further research is needed to identify and characterize risk factors for these malignancies.

Based on the results of this study, future research could further explore the exact cause factors of brain and CNS cancers across different genders, age groups, and national development levels. It is imperative to explore the risk factors for brain and CNS cancers, as addressing this knowledge gap is crucial for analyzing disease burden and improving prevention and control strategies. Additionally, future studies should investigate the application of emerging technologies, such as artificial intelligence and telemedicine, in the prevention, diagnosis, and management of brain and CNS cancers. These innovations hold significant potential, particularly in developing countries, to enhance the accuracy of disease screening and diagnosis, optimize healthcare resource allocation, and ultimately reduce health disparities.

## Conclusion

Overall, despite declining ASMR and ASDR for brain and CNS cancers in China, the disease burden remains substantial compared with that in developed countries. The future disease burden in China is projected to increase due to its large population, aging demographics, and relatively undeveloped prevention and treatment strategies. The Chinese government should prioritize high-burden populations (pediatric, middle-aged and elderly patients). It is necessary to strengthen the early screening and treatment of children and reduce carcinogenic exposure among parents of reproductive age and newborns. In addition, healthcare resource allocation should focus particularly on middle-aged and elderly patients. Exploration of advanced diagnostic and therapeutic technologies is essential, meanwhile collaborate with developed nations to share disease-related data. Public health education should be strengthened to promote awareness of early diagnosis and treatment. Furthermore, increased investment in healthcare resources is needed to train more neurological specialists and improve public health infrastructure. Adopting effective cancer prevention and management practices from developed countries, while considering China’s national context, is essential for addressing this significant public health challenge.

## Supplementary Information

Supplementary Material 1.

## Data Availability

The datasets generated during the current study are available in the Global Health Data Exchange query tool (https://vizhub.healthdata.org/gbd-results/).

## Abbreviations

CNS: Central nervous system
US: the United States
UK: the United Kingdom
DALYs: Disability-adjusted life years
GBD: Global Burden of Disease
EAPC: Estimated annual percentage changes
SII: Inequality Slope Index
ASRs: Age-standardized rates
ASPR: Age-standardized prevalence rate
ASIR: Age-standardized incidence rate
ASMR: Age-standardized mortality rate
ASDR: Age-standardized DALYs rate
NCI: National Cancer Institute
NHS: National Health Service
TFR: Total fertility rate
CI: Confidence interval
UI: Uncertainty interval
SDI: Socio-demographic index
ARIMA: Autoregressive integral moving average
AR: Autoregressive
MA: Moving average
