# Supplementary material for: The comparative burden of brain and central nervous system cancers from 1990 to 2021 between China, the United States, the United Kingdom, and Japan
Source: BMC Public Health. 2025 Aug 6;25:2670. doi: 10.1186/s12889-025-23982-9 (PMC12326625; doi:10.1186/s12889-025-23982-9)
Supplement: Supplementary file 1 — Supplementary Material 1. [file 12889_2025_23982_MOESM1_ESM.docx]

**Additional File 1**


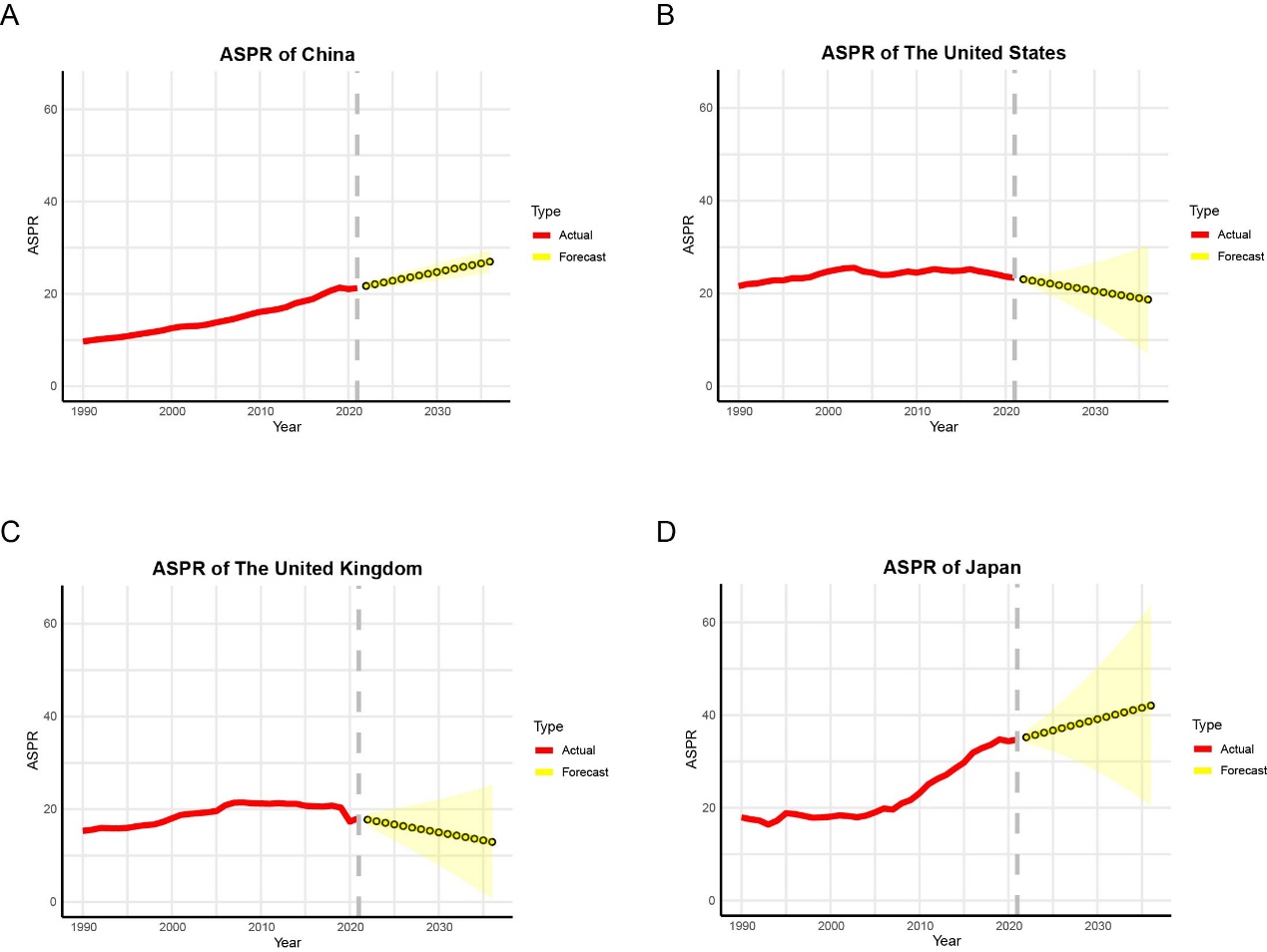


**Fig. S1. Predictive analysis of ASPR in four countries over the next 15 years.**

(A). Temporal trend of ASPR for brain and CNS cancers in China from 2022 to 2036; (B) ASPR forecast for brain and CNS cancers in the United States over the next 15 years; (C). Projected temporal trends in ASPR of brain and CNS cancers in the United Kingdom from 2022 to 2036; (D) Temporal trend in ASPR for brain and CNS cancers in Japan over the next 15 years.


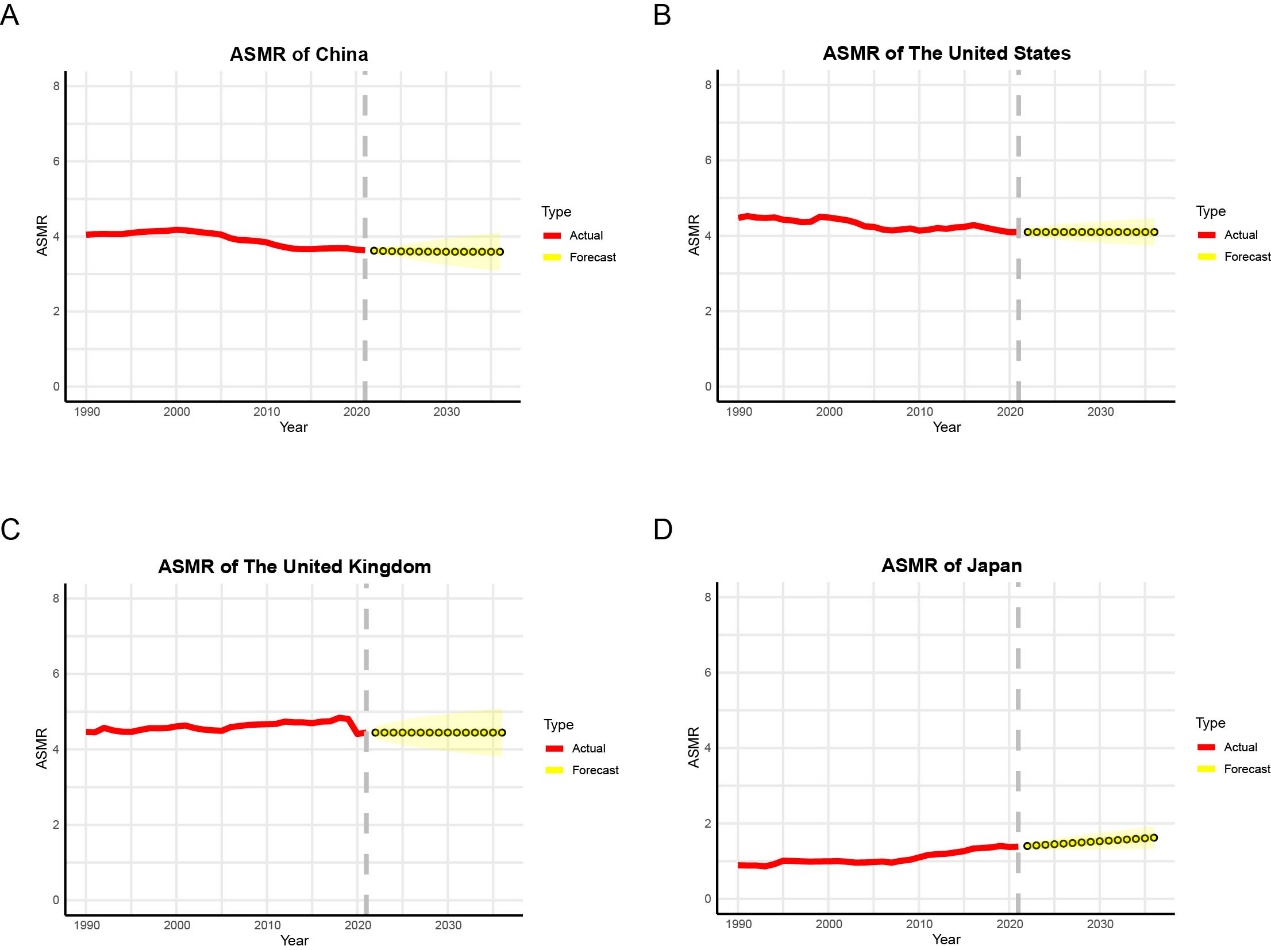


**Fig. S2. Predictive analysis of ASMR in four countries over the next 15 years.**

(A). Temporal trend of ASMR for brain and CNS cancers in China from 2022 to 2036; (B) ASMR forecast for brain and CNS cancers in the United States over the next 15 years; (C). Projected temporal trends in ASMR of brain and CNS cancers in the United Kingdom from 2022 to 2036; (D) Temporal trend in ASMR for brain and CNS cancers in Japan over the next 15 years.


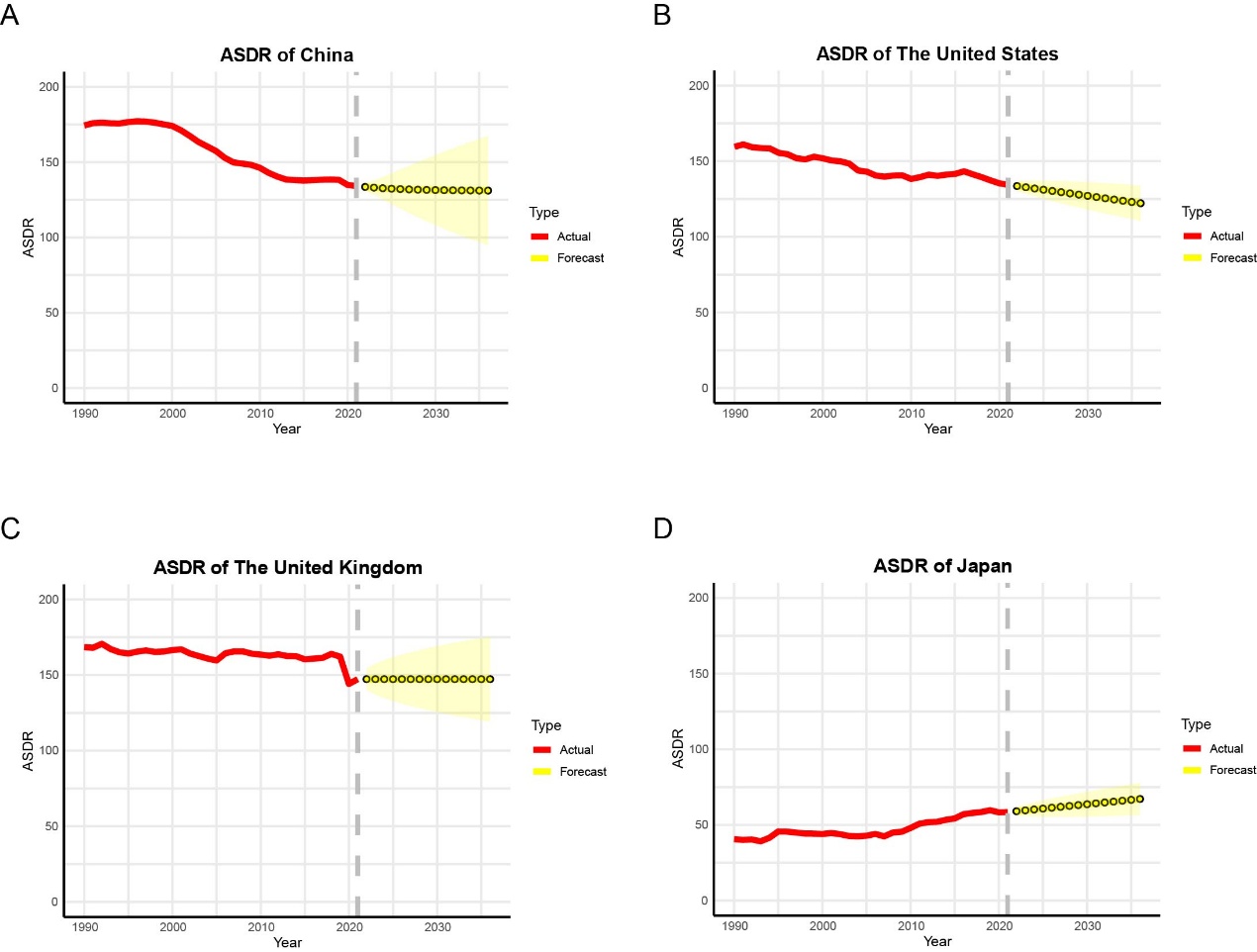


**Fig. S3. Predictive analysis of ASDR in four countries over the next 15 years.**

(A). Temporal trend of ASDR for brain and CNS cancers in China from 2022 to 2036; (B) ASDR forecast for brain and CNS cancers in the United States over the next 15 years; (C). Projected temporal trends in ASDR of brain and CNS cancers in the United Kingdom from 2022 to 2036; (D) Temporal trend in ASDR for brain and CNS cancers in Japan over the next 15 years.
